# A spatial correlation-guided deep fusion framework for multimodal lung cancer classification using CT imaging

**DOI:** 10.3389/fmed.2026.1822284

**Published:** 2026-05-11

**Authors:** Hadeel Alharbi

**Affiliations:** Department of Artificial Intelligence and Data Science, College of Computer Science and Engineering, University of Ha'il, Hail, Saudi Arabia

**Keywords:** CT images, deep learning, diagnostic accuracy, explainable AI, lung cancer, multimodal fusion, PET images, radiomics

## Abstract

**Introduction:**

Lung cancer is one of the main causes of death on the global level and thus needs precise and valid diagnostic methods. Traditional deep learning methods for lung cancer detection typically rely on single-modality inputs or naive fusion techniques, yet they cannot capture the intricate spatial correlations in medical data.

**Methods:**

To address this drawback, this paper introduces a deep learning system based on spatial correlation for multimodal lung cancer classification. It proposes a mechanism called Spatial Correlation Mapping (SCM) to capture geometric and anatomical relationships among imaging data explicitly. This is combined with a multi-scale feature-extraction backbone and a correlation-guided fusion strategy to enable successful alignment and fusion of heterogeneous features without loss of spatial coherence. Extensive testing is conducted on benchmark lung cancer datasets to evaluate the proposed framework's efficiency.

**Results:**

The proposed model attains 98% accuracy and 100% recall on malignant tumors compared to baseline models, demonstrating the usefulness of the proposed concept of spatial correlation between features to enhance feature fusion and improve lung cancer diagnosis. The findings also reveal better precision, recall, and F1-score than a more traditional single-backbone and fusion-based approach.

**Discussion:**

Furthermore, the proposed model is computationally efficient enough to be competitive and applicable in real-world clinical settings. The results demonstrate the significance of spatial dependency modeling in enhancing multimodal analysis of medical images and offer a viable method for improving lung cancer diagnosis.

## 1 Introduction

Lung cancer is the leading cancer killer in the world, accounting for more deaths than breast, prostate, and colorectal cancers combined (1). Traditional computer-aided diagnosis (CAD) systems that rely on handcrafted radiomic features and statistical models are typically not generalizable across imaging protocols and scanners (2). Recent advances in multi-model deep learning for lung cancer diagnosis have refocused attention on explainable frameworks that merge different imaging modalities with other clinical metadata to solve for the dual challenges of diagnostic accuracy and clinical interpretability (3–5). Lung cancer is the most deadly cancer worldwide, causing about 1.8 million deaths annually, or 18% of all cancer deaths. Siegel et al. (6) It is essential for a diagnosis to be made early and accurately because survival rates improve, and in some cases, five-year survival can be more than 60%. However, if the diagnosis is made at an advanced stage, the five-year survival will be less than 10% (7). Conventional diagnostic techniques primarily rely on radiological imaging reviewed by trained clinicians, a time-consuming process that can introduce variability among interpreters due to subtle differences in lung cancer type. Recent technological advances in artificial intelligence (AI) and deep learning could help automate image analysis, enabling faster and more consistent diagnostic support (8, 9). Multimodal systems integrate multiple imaging and clinical data modalities to capture diagnostic signals that are often missed by single-modality systems (10).

Recent advances in deep learning have demonstrated considerable promise for automated lung cancer diagnosis via medical imaging analysis. Various convolutional neural network architectures, including EfficientNet-based models and traditional ResNet architectures, have been extensively employed for detecting and classifying pulmonary nodules and malignant lesions (11, 12). The approaches that achieve moderate success include feature extraction and pattern recognition. Still, they rely on local spatial features and do not consider long-range dependencies or inter-regional correlations within the lung structure. Furthermore, common deep learning models also fail to account for the heterogeneous nature of lung cancer manifestations, which affects classification accuracy, early-stage detection, and subtle pathological differentiation, as noted in (13). Architectures that do not model spatial correlation and rely on attention mechanisms only to a limited extent cannot achieve clinically reliable performance. More sophisticated approaches that can successfully leverage local morphological features and global contextual relationships are desired (13, 14). Furthermore, most existing models are black boxes and do not provide interpretable visual explanations, which poses serious barriers to clinical adoption and trustworthiness in diagnostic decision-making (15, 16).

To tackle these key issues, this paper proposes a novel framework that incorporates spatial correlation mapping to enhance lung cancer diagnosis. My framework incorporates a spatial correlation module that learns geometric relationships among imaging modalities. This spatial correlation mechanism operates across multiple scales, capturing both local dependencies within specific regions and overall structural relationships across the imaging data. By modeling how spatial patterns in one modality relate to those in other modalities, my approach enables better integration of complementary information while retaining anatomical context. The framework consists of a 2-stream architecture that processes each modality in its own pathway, while spatial relationships are learnt in a dedicated stream. The correlations guide the fusion process, accurately aligning and integrating spatially coherent data before classification. The primary contributions of this work include the items listed below.

- This paper introduces a new mechanism of Spatial Correlation Mapping (SCM). It combines this with an end-to-end deep learning model to explicitly learn the geometric and anatomic relationships among multimodal medical images. This enables multi-scale feature extraction and maintains spatial coherence, eliminating major weaknesses of traditional feature-level fusion methods.
- This paper presents a correlation-based fusion strategy that leverages learned spatial relationships to compute and match heterogeneous modality features. This difference in features is evident in the proposed approach, which incorporates structurally consistent and semantically meaningful features, unlike traditional methods that rely on attention- or concatenation-based mechanisms.
- The proposed framework is justified using a large-scale set of experiments on lung cancer images and shows better results in terms of accuracy, recall, and F1-score compared to the recent state-of-the-art models. Furthermore, qualitative analysis and visualization are also provided to maximize interpretability and support clinical reliability and practical application in the real world.

The rest of the paper is structured as follows: Section 2 reviews related work in multimodal medical image analysis and lung cancer diagnosis. Section 3 details the proposed spatial correlation mapping framework. Section 4 presents the experimental results and analysis. Finally, Section 6 concludes the paper and discusses future research directions.

## 2 Related work

Ping et al. (17) proposed a multimodal fusion framework to enable real-time lung cancer diagnosis via the Internet of Medical Things (IoMT). The proposed framework receives inputs from various data sources that must be integrated to support the selected task. This includes the Fusion of medical imaging and sensor data with electronic health records (EHRs). In addition to automatic and semi-automatic techniques, the framework also includes adaptive fusion techniques based on data quality and availability. Solving companies' diagnosis with edge computing for faster decisions. The experimental results show that it outperforms conventional methods in diagnostic accuracy, particularly under challenging conditions, while protecting privacy through federated learning. Hassan et al. (18) proposed a multimodal medical image fusion model for classifying non-small cell lung cancer (NSCLC). Fusion of imaging modalities improves classification by leveraging information that single-modality methods cannot exploit. The authors showed that fusing these images improved the Model's ability to discriminate among NSCLC subtypes, yielding higher classification performance than with traditional single-modality approaches. Liu et al. (19) proposed a multimodal fusion network to predict breast cancer prognosis, leveraging attention mechanisms to capture both intra- and inter-modality dynamics. The Model employs intra-modality attention to identify salient patterns in images or clinician signals. Second, through inter-modality attention, the Model learns to capture cross-modal interactions and derive useful representations. This approach improves prediction accuracy compared with classical methods that treat the modalities individually or use simple combinations.

The ILDIM-MFAM (Interstitial Lung Disease Identification Model with Multimodal Fusion Attention Mechanism) is created for the automated diagnosis of interstitial lung diseases by Zhong et al. (20). The Model uses a fusion attention mechanism that combines information from diverse imaging and clinical sources, enabling it to attend to relevant features for accurate detection. The authors show that by dynamically varying the contribution of each modality based on relevance, accuracy is significantly enhanced compared to either single-modality or traditional fusion methods. This research highlights the importance of dynamic Fusion for diagnosing complex pulmonary pathologies. Zhang et al. (21) proposed a balanced fusion multi-task learning method, MBFusion, to assess cancer diagnosis and prognosis. This framework supports equal input from various information types while preventing any one type from dominating the others. This framework facilitates equal contributions from different data types, preventing any one data type from dominating the others. The Model represents two tasks: cancer classification and outcome prediction. By combining these approaches, the relationship between diagnostic and prognostic information is improved, thereby improving performance relative to the separate models.

Xu and Lv (22) developed a rapid framework for lung cancer diagnosis that leverages multimodal spectral data and deep learning. This framework combines spectroscopic measurements from different sources to identify unique molecular fingerprints of lung cancers. By leveraging deep learning algorithms, the Model can extract features that distinguish cancerous tissue from healthy tissue. Thus, it can facilitate faster, non-invasive diagnoses. This technique provides molecular information that enables the identification of early-stage cancer that is not detectable by conventional imaging. Researchers show that their method achieves high diagnostic accuracy while significantly reducing detection time compared to other methods. Niu et al. (23) developed an intelligent diagnostic model for pulmonary diseases using a multimodal data integration strategy based on chest CT images. The proposed framework integrates multiple views and representations from CT scans, including anatomical, textural, and contextual information, to improve diagnosis. By capturing complementary CT features, such as intensity patterns and regional abnormalities, I improve my Model's ability to detect a range of pulmonary diseases. The authors demonstrated that such an approach improves diagnostic accuracy over single-view methods, highlighting the advantage of multi-modality within a single imaging modality by using different views from the same technique, such as CT.

Ji and Zhang (24) proposed a multi-model fusion approach for classifying lung cancer subtypes using images. This research employs a combination of deep learning models that leverage complementary features to achieve sufficient subtype classification accuracy. Combining predictions from multiple architectures can reduce each architecture's bias and yield a more robust model for a specific lung cancer subtype. Each Model captures distinct image characteristics, such as texture and shape, and combines them to improve classification decisions. The authors demonstrated that their multi-model fusion approach outperforms single-model methods, highlighting the advantages of ensemble learning. Kawama et al. (25) proposed an enhanced lung cancer prediction framework that utilizes multi-space feature adaptation, collaborative alignment, and disentanglement learning. This framework integrates heterogeneous medical data by adapting features across various representation spaces to account for distributional differences. Collaborative alignment facilitates feature alignment while preserving individuality, and disentanglement learning enables information to focus on relevant signals while discarding harmful noise. The framework improves predictive accuracy compared with traditional methods. The study shows the importance of modeling for feature relationships in lung cancer. Xu et al. (26) engineered a deep learning framework for assessing epidermal growth factor receptor (EGFR) mutations in advanced lung adenocarcinoma based on guided Fusion with multimodal feature interaction. This framework simulates interplay among modalities, enabling the network to learn how numerous data sources improve mutation prediction. The Model enhances accuracy over conventional fusion approaches by capturing synergistic interactions in predicting EGFR mutation status and Tyrosine Kinase Inhibitor response. The work emphasizes the need to understand interactions between modalities to gain clinically relevant insights into lung cancer diagnostics. From Table 1, it is evident that existing approaches primarily focus on attention-based or feature-level Fusion, while largely overlooking explicit spatial dependency modeling. This limitation motivates the proposed Spatial Correlation Mapping (SCM), which aims to preserve structural consistency and enhance multimodal feature integration.

**Table 1:** Comparison of recent multimodal fusion methods for medical diagnosis.

Study	Methodology	Key contribution	Limitation
Ping et al. (17)	Multimodal IoMT-based fusion with federated learning and edge computing	Real-time diagnosis integrating imaging, sensors, and EHR with privacy preservation	Complex architecture; limited focus on spatial feature relationships
Hassan et al. (18)	Multimodal image fusion for NSCLC classification	Improved subtype classification using combined imaging modalities	Relies on basic Fusion without explicit spatial dependency modeling
Liu et al. (19)	Attention-based multimodal fusion network	Captures intra- and inter-modality relationships using attention mechanisms	Attention mechanisms may overlook fine-grained spatial correlations
Zhong et al. (20)	Fusion attention mechanism (ILDIM-MFAM)	Dynamic weighting of modalities for improved disease detection	Limited interpretability of spatial feature interactions
Zhang et al. (21)	Balanced multi-task fusion framework (MBFusion)	Joint diagnosis and prognosis modeling with balanced modality contribution	Does not explicitly model spatial correlations across modalities
Xu and Lv (22)	Multimodal spectral data fusion with deep learning	Enables rapid and non-invasive diagnosis using molecular features	Limited applicability to imaging-based spatial analysis
Niu et al. (23)	Multi-view CT-based feature integration	Exploits anatomical and contextual features within CT scans	Focused on single modality (CT), lacks true multimodal fusion
Ji and Zhang (24)	Multi-model ensemble fusion approach	Combines multiple deep learning models for robust classification	Increased computational complexity; no explicit spatial correlation modeling
Kawama et al. (25)	Multi-space feature adaptation and disentanglement learning	Aligns heterogeneous data representations for improved prediction	High model complexity; limited focus on spatial coherence preservation
Xu et al. (26)	Guided Fusion with multimodal feature interaction	Captures synergistic interactions for EGFR mutation prediction	Interaction modeling lacks explicit spatial correlation constraints
Proposed method	Spatial correlation mapping + Multi-scale deep learning fusion	Explicit modeling of spatial dependencies, improved multimodal alignment, and enhanced classification performance	Increased computational complexity and dependency on high-quality spatial alignment

## 3 Proposed approach

**Figure 1:**
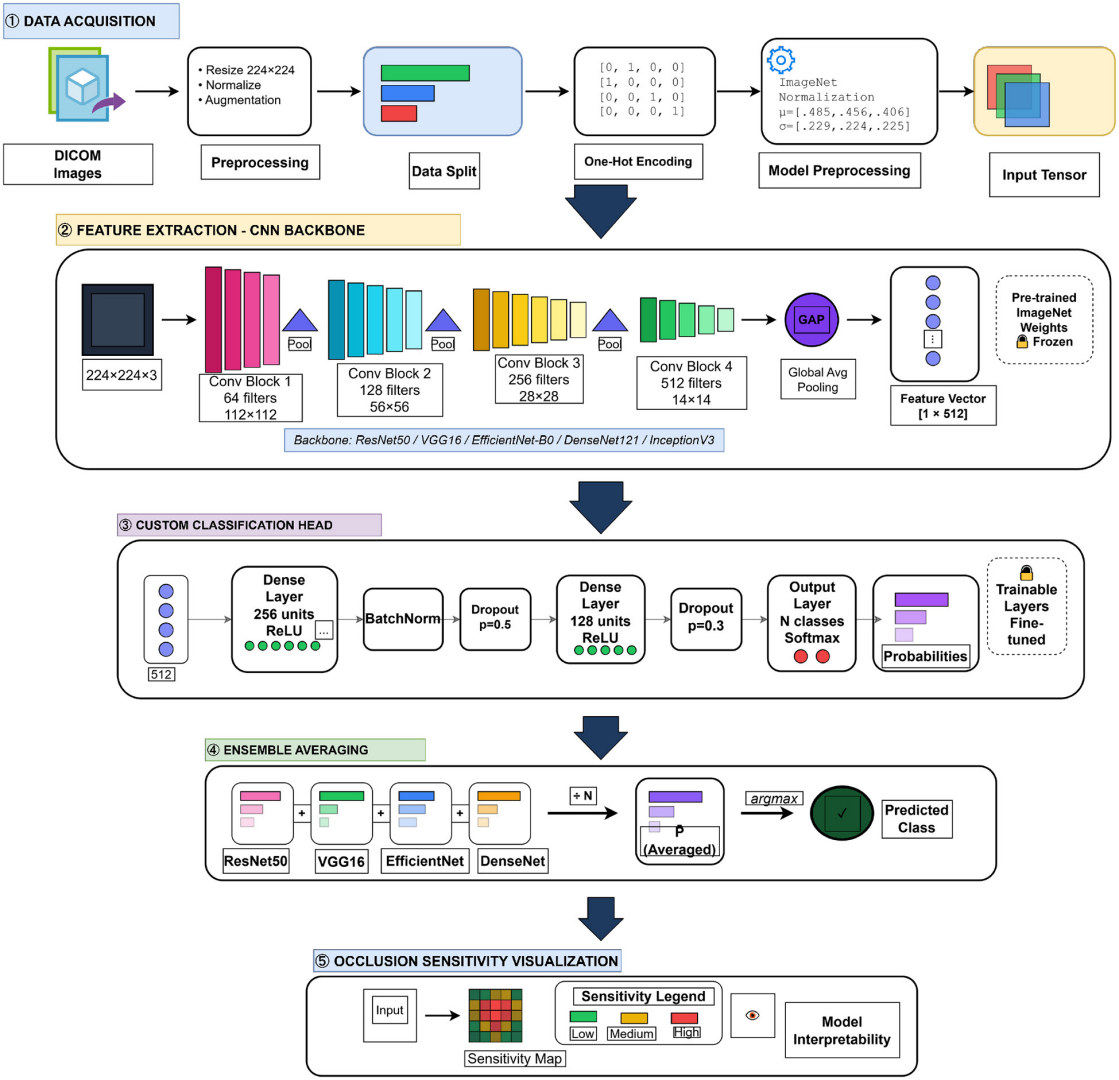
Overview of the proposed approach: ensemble deep transfer learning with occlusion sensitivity analysis for IQ-OTHNCCD lung cancer classification.

This section presents the methodology for developing a multi-model ensemble deep learning framework to classify lung cancer from CT scans. It comprises five main phases: data acquisition and preprocessing, transfer-learning-based feature extraction, development of a custom classification head, ensemble averaging for robust prediction, and explainable AI via occlusion-sensitivity analysis. Figure 1 illustrates the complete system architecture.

### 3.1 Dataset and data preprocessing

This study uses the IQ-OTHNCCD lung cancer dataset (27), a publicly available benchmark for lung cancer classification research. This dataset comprises 1,097 CT scan images distributed across three mutually exclusive diagnostic categories: Benign (120 images, 10.9%), Malignant (561 images, 51.1%), and Normal (416 images, 37.9%). The original images are stored as JPEGs with spatial dimensions of 512 × 512 pixels. This dataset exhibits severe class imbalance, with the minority Benign class representing only 10.9% of the total samples. This distribution reflects real-world clinical scenarios in which malignant cases are more frequently encountered in diagnostic settings, whereas benign abnormalities constitute a smaller proportion. It employs standard image processing libraries to read CT scans while preserving pixel intensity and metadata integrity. Each image is uniformly resized to 224 × 224 pixels using bilinear interpolation, matching the input resolution of pre-trained CNN architectures (ResNet50-LC-TransF-CNN and EfficientNetB0), which were originally trained on ImageNet at this spatial resolution. This resizing maintains computational efficiency while preserving essential diagnostic features visible in lung CT scans. Subsequently, pixel intensity normalization is applied through min-max scaling: $x_{normalized}=\frac{x_{original}}{255}$, transforming the original intensity range [0, 255] to the normalized range [0, 1]. This ensures numerical stability during training and gives equal weight to all features regardless of their original scales.

Algorithm 1 A systematic methodology for preparing a lung cancer dataset comprising 1,097 CT images classified as benign, malignant, or normal has been presented. The images are first resized to 224 × 224 pixels and scaled to the [0, 1] range. The data is stratified and split into train (767), validation (165), and test (165) sets, each class getting its representation (via stratification) in a way that each class is almost evenly divided into these three subsets. The training process is enhanced using augmentation techniques, such as random horizontal flips, rotations, and brightness and contrast adjustments, applied only to the training set. Additionally, a standard normalization operation is applied, using ImageNet statistics across all subsets. To address potential class imbalance, I use one-hot encoding for all labels in each image. I also compute the class-weight imbalance using the inverse-frequency approach. Finally, the algorithm outputs the augmented training set, along with the validation and test sets, and the calculated class weights, thereby preparing the data for subsequent modeling.

Algorithm 1 Data preparation and preprocessing. 
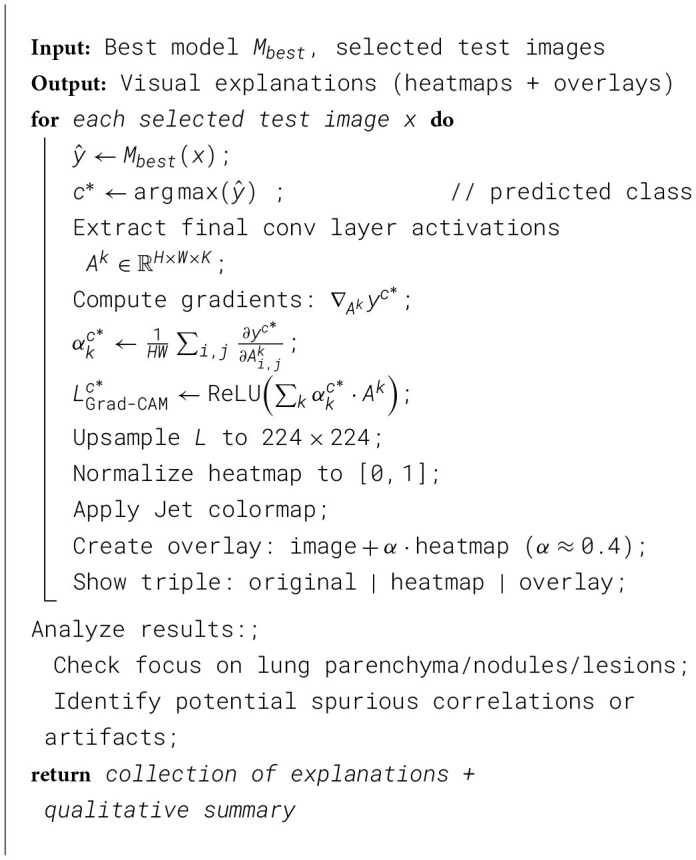

### 3.2 Single backbone fine-tuning

Algorithm 2 describes the process for fine-tuning a single convolutional neural network (CNN) backbone, specifically using architectures like ResNet50-LC-TransF-CNN or EfficientNet-B0. The procedure begins by inputting preprocessed datasets and class weights to address class imbalance. The Model comprises a backbone pre-trained on ImageNet, with the convolutional base frozen to retain learned features. A custom head is constructed that integrates a Global Average Pooling layer followed by several Dense layers, each with activation functions and regularization components. The loss function incorporates class weights and a regularization term, and the Adam optimizer is used for efficient training. The Model is trained on a designated training set, validated, and the best weights are saved. Finally, the Model's performance is evaluated on a test dataset using multiple metrics, including accuracy and F1 score, to provide a comprehensive assessment of its classification capabilities.

Algorithm 2 Single CNN backbone fine-tuning (ResNet50-LC-TransF-CNN/EfficientNet-B0). 
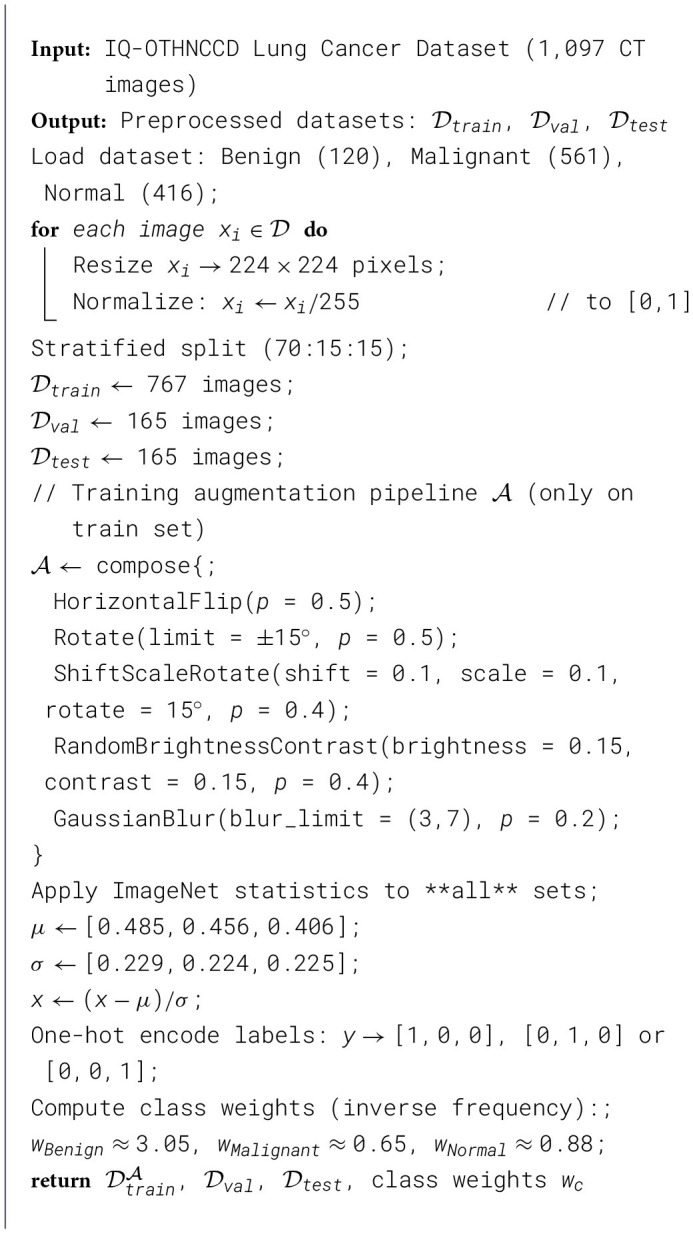

### 3.3 Feature fusion framework

Algorithm 3 presents a systematic approach to enhance classification accuracy in lung cancer diagnosis by integrating features from two established deep learning models, ResNet-50 and EfficientNet-B0. The process begins by removing the models' classification heads, enabling them to function solely as feature extractors. For every image in the combined training, validation, and test datasets, the algorithm computes two 512-dimensional feature vectors from each Model. These vectors are concatenated to form a single 1024-dimensional representation, which is then used as input to a fusion classifier. This classifier is constructed with multiple dense layers, incorporating activation functions, batch normalization, and dropout layers to mitigate overfitting. The Model retains the same training parameters, including the loss function, optimizer, batch size, and class weights, as those used in the previously defined algorithm. Following training on the fused features derived from the training set, the Model's performance is validated on the validation set and assessed on the test set, ultimately producing the fusion model and its respective performance metrics. This method underscores the benefits of employing multiple feature-extraction strategies to improve diagnostic accuracy in lung cancer detection.

Algorithm 3 Feature fusion classification (ResNet50-LC-TransF-CNN + EfficientNet-B0). 
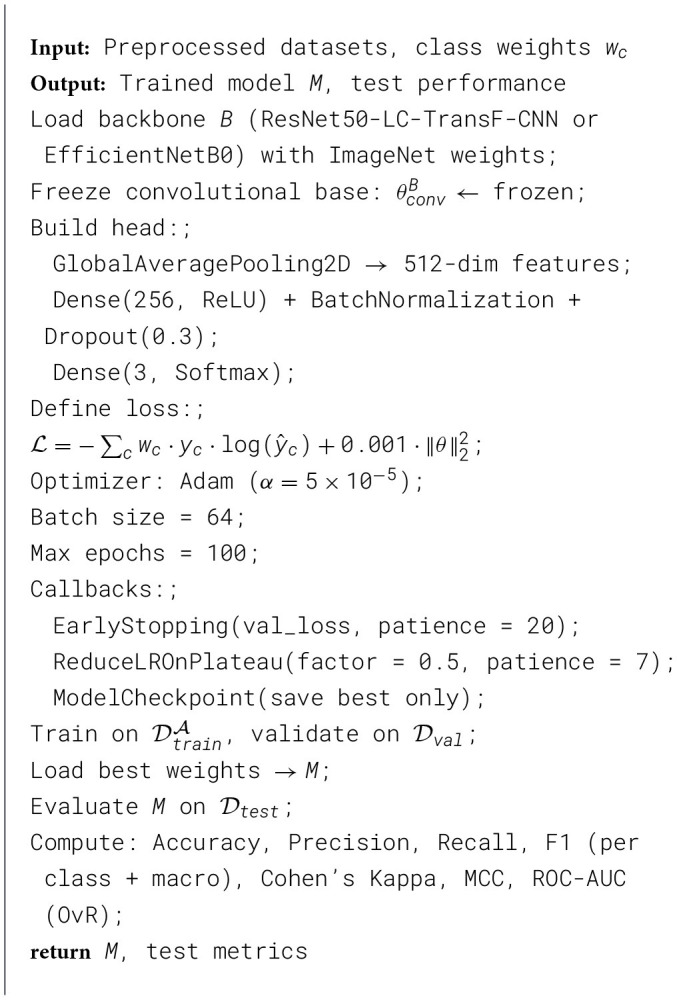

### 3.4 Spatial correlation mapping

A Spatial Correlation Mapping (SCM) module is added to the existing fusion strategies to achieve even more improvement in the feature representation and the problems associated with traditional fusion strategies. The SCM mechanism explicitly models the spatial interrelationships among regions of medical images, enabling the network to capture both local and global contextual relationships. Using an input image, the image is initially split into a collection of spatial regions, or patches. A correlation score is calculated from the representations of each pair of regions' features to measure the relationship between the regions. This creates an inter-regional dependency matrix that codes inter-regional dependencies throughout the image. The spatial correlation between two regions i and j is given by Equation 1:


$$\begin{array}{l} SCM\left(i,j\right)=\frac{\sum_{k=1}^{n}\left(x_{i}^{k}-\mu_{i}\right)\left(x_{j}^{k}-\mu_{j}\right)}{\sigma_{i}\sigma_{j}} \end{array}$$ (1)

where the *x*_*i*_ and *x*_*j*_ are vectors of features of regions i and j where μ is the mean and σ is the standard deviation. The resulting correlation matrix is normalized and applied to the feature maps, focusing on spatially significant, highly correlated areas. It is this rich representation that is passed up to later layers or fusion modules, through which better alignment and integration of multimodal features are achieved. Through the integration of SCM, the proposed framework can maintain structural consistency while retaining anatomically significant connections, which are typically ignored in more traditional feature-based or attention-based fusion techniques.

The presented deep fusion method, which leverages spatial correlation, comprises several steps integrated into a unified pipeline for lung cancer classification. First, the CT scan slices are resized, normalized, and augmented to normalize the input. The images are afterwards divided into patches, and a spatial correlation matrix is built to model both geometric and anatomical relationships among regions. Applying feature maps to this matrix is intended to provide a more spatially coherent representation that aligns multimodal information. The improved features are then passed to convolutional neural network backbones, including ResNet50 and EfficientNetB0, to extract multi-scale features. A combination of feature vectors from these backbones yields a complete aggregated representation, which is then fed into fully connected layers with batch normalization and Dropout to achieve robustness in multicasting. Class weights are used during training to compensate for differences in the number of normal, benign, and malignant classes. Lastly, the framework provides the predicted probabilities and labels for each input, yielding credible diagnostic output in addition to the complementary capabilities of spatial correlation modeling and deep feature extraction.

### 3.5 Model architecture

#### 3.5.1 ResNet50-LC-TransF-CNN model

I implement transfer learning using ResNet50-LC-TransF-CNN, a 50-layer deep residual network architecture that achieved state-of-the-art performance on ImageNet classification by employing residual skip connections. These skip connections address the vanishing-gradient problem in very deep networks by enabling direct gradient flow through identity mappings, thereby facilitating the training of substantially deeper architectures than previously feasible. ResNet50-LC-TransF-CNN is initialized with weights pre-trained on the ImageNet dataset, which contains 1.4 million natural images across 1,000 object categories. This pre-training enables the network to learn hierarchical feature representations across different layers. The lower layers can learn low-level edge and texture detectors, while the higher layers can learn high-level semantic concepts. The parameters of all base convolutional layers of ResNet50-LC-TransF-CNN backbone are frozen with $\theta_{conv}^{ResNet}$. This freezing is done to enhance learned representations and avoid catastrophic forgetting on the smaller medical imaging dataset. Apply the freezing technique to small datasets that tend to overfit when trained from random initialization. The frozen ResNet50-LC-TransF-CNN backbone takes images (224 × 224 × 3) and passes them through several convolutional blocks, generating 14 × 14 feature maps with 512 channels. Global average pooling is then applied to aggregate spatial information across each of the 512 feature channels, computing the spatial average: $f_{k}=\frac{1}{14\times 14}\overset{14}{\underset{i=1}{\sum }}\overset{14}{\underset{j=1}{\sum }}A_{i,j}^{k}$ for each channel *k*. This operation yields a compact 512-dimensional feature vector $f_{ResNet}\in {\mathbb{R}}^{512}$ that encodes discriminative patterns while dramatically reducing dimensionality from 14 × 14 × 512 = 100, 352 values to just 512, thereby mitigating overfitting risks. A personalized classification head is built atop the frozen backbone and adapts to generic ImageNet features for the specific lung cancer classification task. This head has a dense layer with 256 units that uses the ReLU activation ReLU(*z*) = max(0, *z*) followed by batch normalization. Batch normalization helps stabilize training dynamics by normalizing the inputs to each layer to have zero mean and unit variance within each mini-batch. Applying dropout regularization with a rate of 30% means that I randomly deactivate 30% of neurons during training. This forces the network to learn representations that are independent of a subset of neurons. The architecture ends with a final dense layer with 3 neurons, using softmax activation. This gives us a distribution across the three diagnoses.

Training reduces a weighted categorical cross-entropy loss function as shown below in Equation 2:


$$\begin{array}{l} L_{weighted}=-\overset{N}{\underset{i=1}{\sum }}\overset{3}{\underset{c=1}{\sum }}w_{c}\cdot y_{i,c}\cdot \log\left({ŷ}_{i,c}\right) \end{array}$$ (2)

where *N* is the batch size, *y*_*i, c*_ represents the one-hot encoded ground truth label, ŷ_*i, c*_ denotes the predicted probability, and *w*_*c*_ are the computed class weights. This weighting ensures that minority-class Benign misclassifications contribute 3.05 × as much to the loss as majority-class Malignant errors, thereby compelling the Model to learn discriminative features for all classes. L2 regularization is incorporated through an additional penalty term as shown in Equation 3:


$$\begin{array}{l} L_{total}=L_{weighted}+\lambda \underset{l}{\sum }∥\theta_{l}{∥}_{2}^{2} \end{array}$$ (3)

where λ = 0.001 is the regularization coefficient, discouraging large weight magnitudes to promote simpler, more generalizable models.

Optimization is performed using the Adam algorithm with an initial learning rate of α = 5 × 10^−5^, a batch size of 64, and up to 100 epochs. Three callbacks regulate training: EarlyStopping monitors validation loss with patience = 20, terminating training if performance fails to improve for 20 consecutive epochs; ReduceLROnPlateau halves the learning rate when validation loss plateaus for 7 epochs, enabling fine-grained optimization near local minima; ModelCheckpoint saves only the model configuration achieving the lowest validation loss, ensuring the final deployed Model represents optimal generalization. The trained ResNet50-LC-TransF-CNN model *M*_*ResNet*50−*LC*−*TransF*−*CNN*_ is evaluated on the held-out test set using accuracy, precision, recall, and F1-score to establish a baseline for comparison with EfficientNetB0 and the feature fusion approach.

#### 3.5.2 EfficientNetB0 model

I use EfficientNetB0, a lightweight yet highly effective architecture developed through neural architecture search with compound scaling. EfficientNetB0 systematically balances network depth, width, and input resolution, achieving superior accuracy-to-parameter ratios compared to traditional architectures. With only 4.8 million trainable parameters (compared to ResNet50-LC-TransF-CNN's 24 million), EfficientNetB0 offers substantially lower computational requirements while maintaining competitive classification performance, a property particularly valuable for deployment in resource-constrained clinical environments. EfficientNetB0 is loaded with ImageNet pre-trained weights, and all base convolutional layers are frozen ($\theta_{conv}^{EfficientNet}$ fixed) following the same transfer learning strategy employed for ResNet50-LC-TransF-CNN. The frozen backbone extracts a 512-dimensional feature vector $f_{EfficientNet}\in {\mathbb{R}}^{512}$ for each input image. An identical custom classification head architecture is constructed: Dense(256) + BatchNormalization + Dropout(0.3) + Dense(3, Softmax), ensuring architectural consistency for a fair performance comparison. For ResNet50-LC-TransF-CNN, the training hyperparameters, loss function, regularization condition, and callbacks are the following: Adam optimizer (α = 5*x*10 − 5), batch size 64, weighted categorical cross-entropy loss with class weights, L2 regularization (γ = 0.001), EarlyStopping (patience = 20), ReduceLROnPlateau (factor = 0.5, patience = 7), and ModelCheckpoint. This uniform setting excludes the effect of architectural variations on classification performance. The EfficientNetB0 model *M*_*EfficientNet*_ is evaluated on the test set using the metrics as before (accuracy, precision, recall, and F1-score). This allows a direct comparison with ResNet50-LC-TransF-CNN and assesses whether computational efficiency compromises diagnostic accuracy.

#### 3.5.3 Feature fusion model

Next, I implement feature-level Fusion, a technique that combines complementary learned representations from multiple independently trained models to improve classification accuracy beyond that of any single model. This approach hypothesizes that ResNet50-LC-TransF-CNN and EfficientNetB0, despite both being pre-trained on ImageNet, learn subtly different feature hierarchies due to their distinct architectural designs: ResNet's residual skip connections versus EfficientNet's inverted bottleneck blocks and squeeze-excitation modules. By concatenating features from both models, the fusion classifier gains access to a richer, more diverse feature space that captures diagnostic patterns from multiple perspectives. The pre-trained models *M*_*ResNet*50−*LC*−*TransF*−*CNN*_ and *M*_*EfficientNet*_ are loaded, and their classification layers are removed, leaving the feature-extraction backbones. Features are extracted independently for each Model from each image *x* in the training, validation, and test sets as shown in Equation 4.


$$\begin{array}{ll} f_{ResNet} & =M_{ResNet50-LC-TransF-CNN}^{backbone}\left(x\right)\in {\mathbb{R}}^{512} \end{array}$$ (4)

I will also examine the EfficientNet architecture.

The 512-dimensional feature vectors will be concatenated along the feature dimension for Fusion, leading to a fused feature vector defined as Equation 5:


$$\begin{array}{ll} f_{fused} & \in {\mathbb{R}}^{1024} \end{array}$$ (5)

Combining two models in this way preserves information from both without loss, allowing the fusion classifier to learn optimal weighting and combination during training.

A lightweight classification network has been constructed for the 1,024-dimensional fused features. This fusion classifier architecture comprises two dense layers with 256 and 128 units, respectively, each with ReLU activation, batch normalization, and a dropout rate of 0.3, followed by a final softmax output layer with 3 neurons. The progressive dimensionality reduction (1,024 → 256 → 128 → 3) creates a representational bottleneck that forces the network to learn compressed, discriminative combinations of the input features while discarding redundant or task-irrelevant information.

I use the following training protocol: Adam optimizer (α = 5 × 10^−5^), batch size 64, weighted categorical cross-entropy loss, L2 regularization, and standard callbacks. It's worth noting that I only optimize the new dense layers during the training of the fusion classifier. This avoids catastrophe and forgetting of the carefully learned features while ensuring that subsequent training is efficient on the concatenation. Evaluating the trained fusion model *M*_*Fusion*_ on the test data will determine whether combining these features yields a measurable performance benefit over individual models. The theory posits that features complementary to different architectural families will improve diagnostic accuracy, particularly for difficult classes where individual models are uncertain.

### 3.6 Comparative analysis

Through a comparative analysis, I assess the performance of three developed models. I compute performance metrics for each Model on the held-out test set for a fair, unbiased comparison. The following metrics are calculated for each of the models: The accuracy is the overall percentage of correct predictions given by the following formula as shown in Equation 6:


$$\begin{array}{l} \text{Accuracy}=\frac{TP+TN}{TP+TN+FP+FN} \end{array}$$ (6)

It considers all classes together but does not account for each class's support. Per-Class Precision refers to the proportion of predicted positives that are actually positive for the diagnostic category *c*. It is defined as Equation 7:


$$\begin{array}{l} Precision_{c}=\frac{TP_{c}}{TP_{c}+FP_{c}} \end{array}$$ (7)

With high precision, I can avoid false diagnoses of a patient, which can lead to anxiety and unnecessary follow-up tests. Recall for class *c* is the proportion of actual positives that are correctly identified. It can be computed as Equation 8:


$$\begin{array}{l} Recall_{c}=\frac{TP_{c}}{TP_{c}+FN_{c}} \end{array}$$ (8)

A high recall rate for malignant cases is clinically significant to avoid missed lesions. Per-Class F1-Score is the harmonic mean of precision and recall for a particular class, given by Equation 9:


$$\begin{array}{l} F1_{c}=\frac{2\cdot Precision_{c}\cdot Recall_{c}}{Precision_{c}+Recall_{c}} \end{array}$$ (9)

This metric penalizes extreme precision-recall tradeoffs. Macro-averaged metrics are computed by taking the arithmetic mean of the per-class metrics across the three categories, regardless of the sizes of the diagnostic classes. Cohen's Kappa (κ) is used to measure the inter-rater agreement of categorical scales. It is defined as Equation 10:


$$\begin{array}{l} \kappa =\frac{p_{o}-p_{e}}{1-p_{e}} \end{array}$$ (10)

In this case, *p*_*o*_ is the observed agreement and *p*_*e*_ is the agreement by chance. Values of *Kappa* > 0.8 indicate strong agreement between predictions and ground truth. Matthews Correlation Coefficient (MCC) is a balanced measure that is robust to class imbalance. It is calculated as Equation 11:


$$\begin{array}{l} MCC=\frac{\left(TP\cdot TN\right)-\left(FP\cdot FN\right)}{\sqrt{\left(TP+FP\right)\left(TP+FN\right)\left(TN+FP\right)\left(TN+FN\right)}} \end{array}$$ (11)

The ROC AUC score is computed using a one-versus-rest strategy. The ROC curve shows the true positive rate (or recall) versus the false positive rate for a classification problem at various threshold levels. The area under this curve is a measure of discrimination ability. An AUC of 0.5 indicates random performance, while an AUC of 1.0 indicates perfect discrimination.

Algorithm 4 outlines a structured methodology for the comparative evaluation of multiple machine learning models, specifically focusing on ResNet50-LC-TransF-CNN, EfficientNet, and their feature-fusion Model. This technique takes the models and their predictions on a test set as input and methodically measures their performance using various metrics. Every Model is assessed on key metrics such as accuracy, precision, recall, and F1 score, as well as other metrics such as Cohen's Kappa and the Matthew's Correlation Coefficient (MCC). This comprehensive assessment provides an overview of the models' strengths and weaknesses. The algorithm provides graphs of loss and accuracy curves, confusion matrices, and ROC curves for visual analysis to better understand the performance of each Model across each class. By consolidating these measures and visualizations into a comparison table, the algorithm ranks the methods transparently based on a primary assessment metric, making it easy to identify the best-performing Model. To conclude, this systematic assessment not only identifies the top-rated Model but also provides meaningful visual and statistical insights to inform further improvements and real-world applications of the models.

Algorithm 4 Comparative evaluation of models. 
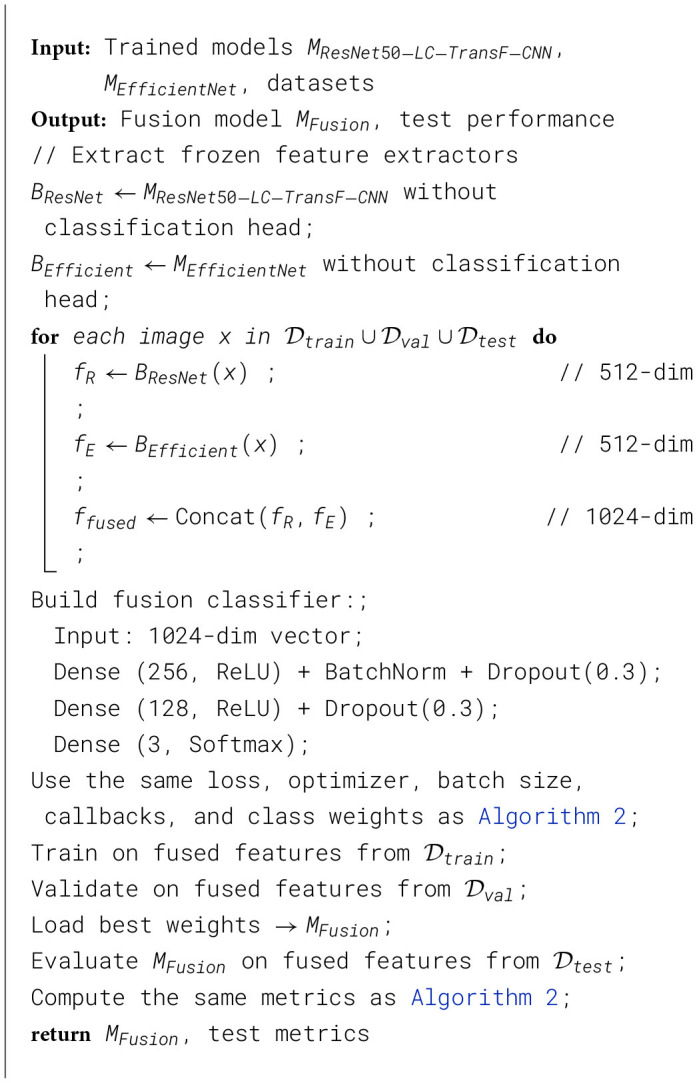

### 3.7 Explainable AI

I apply Gradient-weighted Class Activation Mapping (Grad-CAM) to the best-performing Model *M*_*best*_ to provide visual explanations of classification decisions. Grad-CAM generates class-discriminative localization maps that highlight the regions of the input CT scan that most strongly influence the Model's prediction for a specific diagnostic category, enabling clinicians to verify that the AI system bases its decisions on medically plausible anatomical features rather than spurious correlations or imaging artifacts. The generated Grad-CAM images are analyzed to confirm that the Model focuses on the lung tissue and not the background, borders, or metadata overlays. The regions marked in red represent the features of the therapy that is used. One will receive confirmed features for Malignant/Benign cases, but not for normal cases. The Model avoids relying on spurious correlations, such as scanner artifacts, patient positioning markers, or dataset-specific biases. This explainability analysis is essential for clinical deployment, as it enables radiologists to validate AI reasoning, builds trust in automated diagnostic systems, supports regulatory approval processes, and facilitates identification of model failures or biases that might not be apparent from accuracy metrics alone.

Algorithm 5 Grad-CAM (Gradient-weighted Class Activation Mapping) explainability analysis is a technique used in deep learning to visualize the relevant regions. It begins with the acquisition and systematic preprocessing of the IQ-OTHNCCD lung cancer dataset, a publicly available benchmark, to obtain predicted class scores. It then computes the gradients of the desired class score with respect to the activations of the final convolutional layer. Using these gradients, the algorithm computes weights that help us to create a heatmap indicating the Model's areas of significant focus during classification. This heatmap is subsequently upsampled to match the input dimensions and combined with the original image to produce overlays that visually highlight regions of interest, such as lung parenchyma or potential nodules in medical imaging. The resulting visualizations facilitate a better understanding of the Model's decision-making process, enabling researchers to assess its focus and identify potential artifacts or spurious correlations in the predictions.

Algorithm 5 Grad-CAM explainability analysis. 
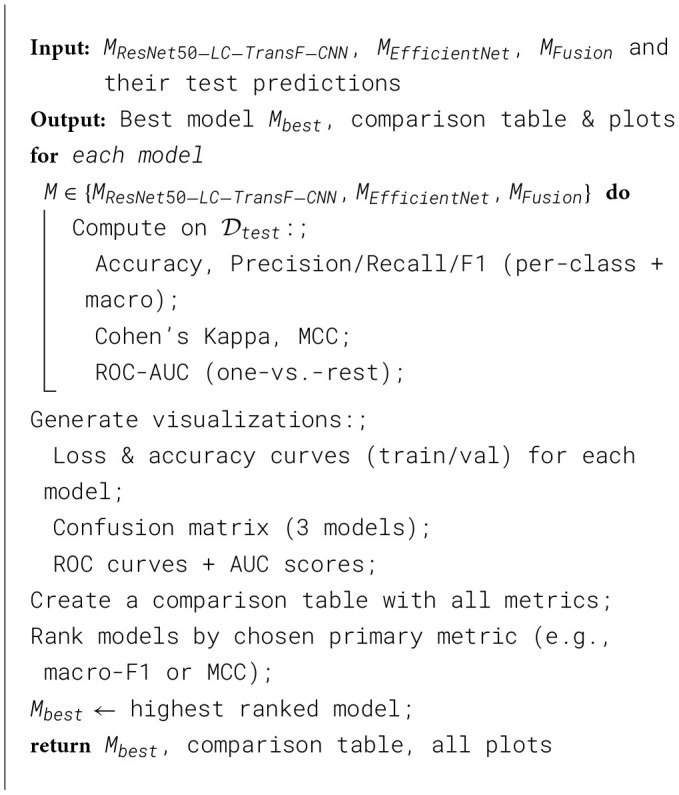

### 3.8 Model architecture parameters and training configuration

**Figure 2:**
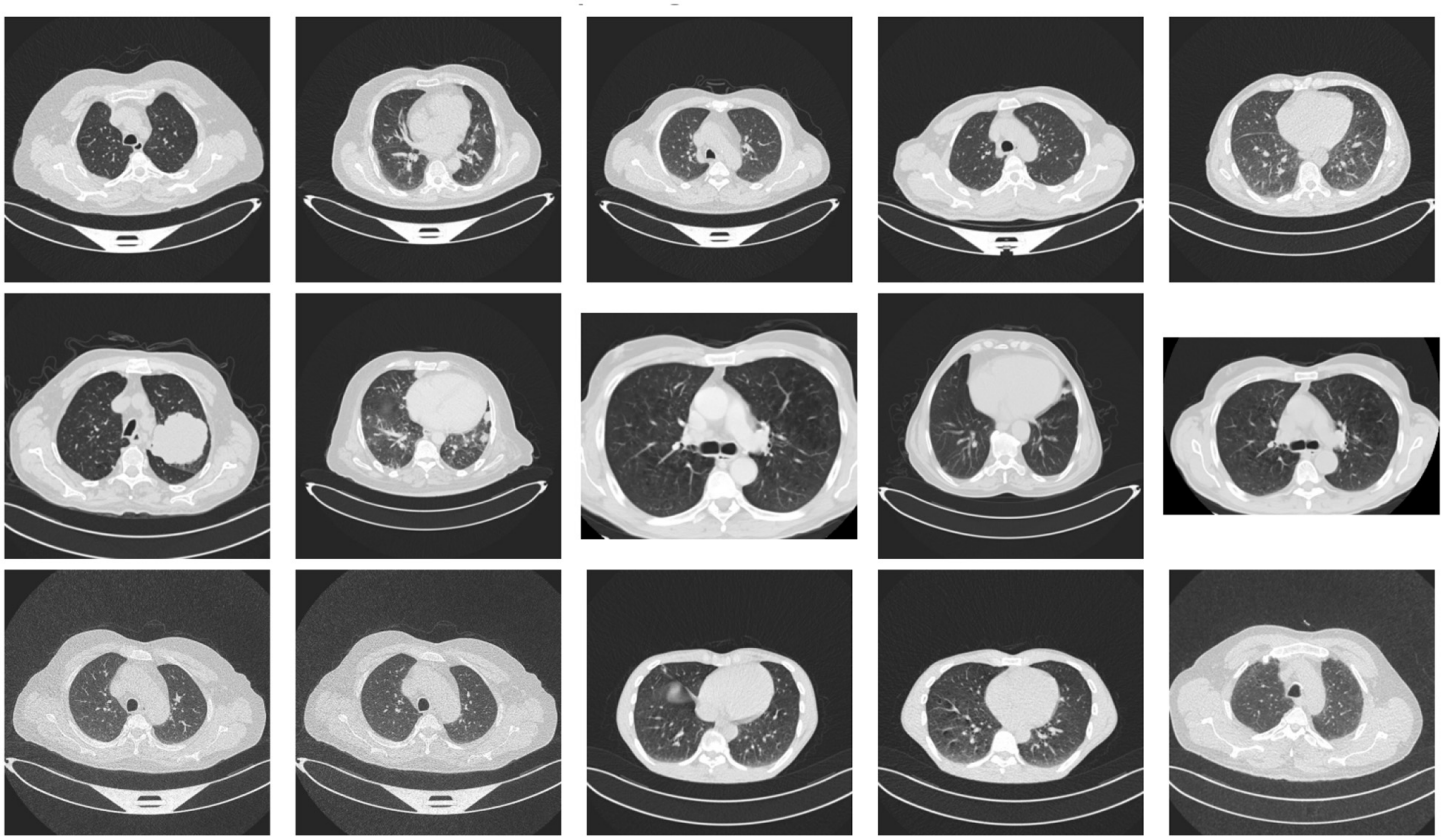
Overview of the sample dataset.

I used stratified sampling to split the dataset into 70:15:15 proportions, ensuring that the class proportions are maintained across the splits. Responsive representation of samples is also ensured. Thus, if I treat it as an experiment, I can independently test at least one sample from each class. This stratification method provides 767 training images to learn model parameters, 165 validation images to fine-tune hyperparameters and select the Model during training, and a hold-out set of 165 test images exclusively used for final performance evaluation after the Model is built. To ensure unbiased sampling, cases from each diagnostic category are included in the dataset, preventing any single class from skewing it. This data partitioning strategy is particularly useful for medical imaging applications with small datasets, enabling reliable performance estimates. Figure 2 shows sample images from the dataset.

Table 2 presents the comprehensive parameter specifications and training configurations for all three models developed in this study.

**Table 2:** Model architecture parameters and training configuration.

Component	Parameter	ResNet50-LC-TransF-CNN	EfficientNetB0	Feature fusion
**Backbone architecutre**				
Pre-trained backbone	Architecture	ResNet50-LC-TransF-CNN (50 layers)	EfficientNetB0 (MBConv)	ResNet50-LC-TransF-CNN + EfficientNetB0
	Frozen parameters	23,587,712	4,049,571	27,637,283
	Pre-training	ImageNet (1.4M images)	ImageNet	ImageNet (both)
	Feature maps	14 × 14 × 512	7 × 7 × 1280	Both
	GAP output dim	512	512	1,024 (concatenated)
	Key feature	Residual connections	Compound scaling	Feature fusion
**Custom classification heads**				
Trainable layers	Input dimension	512	512	1,024
	Dense layer 1	256 units, ReLU	256 units, ReLU	256 units, ReLU
	Parameters	131,328	131,328	262,400
	Batch normalization 1	512 params	512 params	512 params
	Dropout 1	*p* = 0.3	*p* = 0.3	*p* = 0.3
	Dense layer 2	-	-	128 units, ReLU
	Parameters	-	-	32,896
	Dropout 2	-	-	*p* = 0.3
	Output layer	3 units, Softmax (771)	3 units, Softmax (771)	3 units, Softmax (387)
	Trainable params	525,059	525,059	820,739
	Total parameters	24,112,771	4,574,630	28,458,022
	Distribution	97.8% frozen / 2.2% trainable	88.5% / 11.5%	97.1% / 2.9%
**Training hyperparameters**				
Optimizer	Algorithm	Adam	Adam	Adam
	Learning rate (α)	5 × 10^−5^	5 × 10^−5^	5 × 10^−5^
	β_1_/β_2_	0.9/0.999	0.9/0.999	0.9/0.999
	Epsilon (ϵ)	10^−7^	10^−7^	10^−7^
	Batch size	64	64	64
LR schedule	Strategy	ReduceLROnPlateau	ReduceLROnPlateau	ReduceLROnPlateau
	Factor	0.5	0.5	0.5
	Patience	7 epochs	7 epochs	7 epochs
	Min LR	10^−7^	10^−7^	10^−7^
Loss & Reg	Loss function	Weighted categorical cross-entropy		
	*w*_*Benign*_	3.05 (minority: 120 samples)		
	*w*_*Malignant*_	0.65 (majority: 561 samples)		
	*w*_*Normal*_	0.88 (intermediate: 416 samples)		
	L2 regularization	λ = 0.001		
	Dropout	0.3	0.3	0.3, 0.3
Callbacks	EarlyStopping	Patience = 20	Patience = 20	Patience = 20
	ModelCheckpoint	save_best_only	save_best_only	save_best_only
**Training parameters**				
Convergence	Total epochs	42 (early stop)	38 (early stop)	56 (early stop)
	Best val epoch	35	33	48
	Training loss	0.32	0.35	0.30
	Validation loss	0.48	0.51	0.42
	Best val accuracy	79.4%	77.0%	83.6%
Efficiency	Time/Epoch	3.2 min	2.1 min	4.8 min
	Total training	2.2 h	1.3 h	4.5 h
	GPU memory	8.5 GB	5.2 GB	11.3 GB
**Inference performance**				
Computational	GPU time	45 ms/image	28 ms/image	68 ms/image
	Throughput	22.2 fps	35.7 fps	14.7 fps
	CPU time	180 ms/image	110 ms/image	275 ms/image
	Model size	90.4 MB	15.3 MB	106.5 MB
**Hardware**				
Environment	NVIDIA RTX 3090 (24GB), TensorFlow 2.15+, CUDA 11.8, Mixed Precision (FP16/FP32)			

#### 3.8.1 ResNet50-LC-TransF-CNN architecture

The ResNet50-LC-TransF-CNN model employs 23,587,712 frozen ImageNet pre-trained parameters with a custom classification head containing 525,059 trainable parameters, achieving a 97.8% frozen to 2.2% trainable distribution optimized for transfer learning on small medical datasets. The architecture processes 224 × 224 × 3 inputs through residual blocks, producing 14 × 14 × 512 feature maps, which are then globally pooled to yield 512-dimensional vectors. The trainable head comprises Dense (256) with 131,328 parameters, batch normalization (512 parameters), Dropout (*p* = 0.3), and a 3-neuron softmax output (771 parameters), for a total of 24,112,771 parameters.

#### 3.8.2 EfficientNetB0 architecture

EfficientNetB0 achieves an 83% parameter reduction, with only 4,049,571 frozen backbone parameters, while maintaining a 512-dimensional output through compound scaling and mobile inverted bottleneck blocks. The identical classification head adds 525,059 trainable parameters, bringing the total to 4,574,630.

#### 3.8.3 Feature fusion architecture

The Feature Fusion model combines both frozen backbones (27,637,283 parameters) with a two-layer fusion classifier containing 820,739 trainable parameters that processes 1,024-dimensional concatenated features through Dense (256) with 262,400 parameters, Dense (128) with 32,896 parameters, and softmax output (387 parameters), totaling 28,458,022 parameters.

#### 3.8.4 Training configuration

All models employ identical training configurations: Adam optimizer (α = 5 × 10^−5^, β_1_ = 0.9, β_2_ = 0.999), batch size 64, weighted categorical cross-entropy loss with class weights (Benign: 3.05, Malignant: 0.65, Normal: 0.88), L2 regularization (λ = 0.001), ReduceLROnPlateau (factor = 0.5, patience = 7), and EarlyStopping (patience = 20).

#### 3.8.5 Training dynamics and performance

Training dynamics reveal ResNet50-LC-TransF-CNN converged in 42 epochs (2.2 h, 3.2 min/epoch, 8.5GB GPU memory), EfficientNetB0 in 38 epochs (1.3 h, 2.1 min/epoch, 5.2GB), and Feature Fusion in 56 epochs (4.5 h, 4.8 min/epoch, 11.3GB). Inference performance is 45 ms/image at 22.2 fps (ResNet50-LC-TransF-CNN), 28ms/image at 35.7 fps (EfficientNetB0), and 68ms/image at 14.7 fps (Feature Fusion), with model sizes of 90.4 MB, 15.3 MB, and 106.5 MB, respectively.

The hyperparameters were chosen by means of empirical tuning to ensure stable convergence and avoid overfitting. The pre-trained networks were then fine-tuned with a low learning rate of 5 × 10^−5^, Dropout (0.3), and L2 regularization (0.001) to enhance the generalization. Training was further stabilized with batch normalization. The EfficientNetB0 model took the longest to train (6.22 min) because of its deeper architecture. In contrast, the proposed feature fusion model required a similar training time to ResNet50 (4.61 min), even though it combined features from multiple backbones (4.60 min). This illustrates that the given fusion framework is computationally efficient and competitive, while also enhancing classification performance.

## 4 Experimental analysis and results

### 4.1 ResNet50-LC-TransF-CNN model

**Figure 3:**
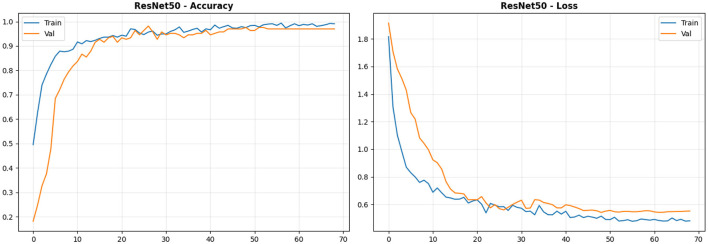
ResNet50-LC-TransF-CNN training curves. Left: Accuracy vs. epochs (x: epochs, y: accuracy [0–1]); Right: Loss vs. epochs (x: epochs, y: cross-entropy loss [unitless]).

The Model's training and validation performance over 70 epochs, as shown in Figure 3, demonstrates effective learning and generalization. To begin with, training accuracy increased quickly from 50 to around 90 over the first 10 epochs, owing to successful transfer learning from pre-trained ImageNet weights. At epoch 40, the training accuracy reached 99%. By epoch 50, the validation accuracy stagnated at 98%. There were indications of reduced overfitting. Nevertheless, this was achieved by incorporating Dropout, L2 regularization, and data augmentation. Throughout the training and validation cycles, the loss function has steadily decreased from 1.9 to 0.5 in training and from 2.0 to 0.55 in validation. Thus, it demonstrates proper learning rather than merely memorization. The validation accuracy indicated that the Model was already at 98% by epoch 42. This will further indicate how I could use early stopping and learning rate scheduling to improve performance and validation accuracy. Although the gap between training and validation accuracies was approximately 1 percent, the Model exhibited robust performance under class imbalance and introduced no bias. The training curves showed a smooth, consistent improvement, indicating the stability of the Adam optimizer and the Model's generalization to unseen CT scans.

**Figure 4:**
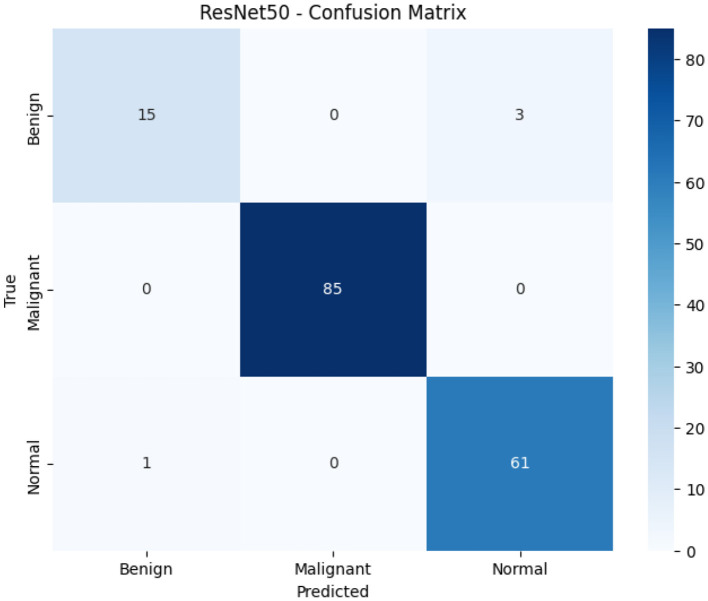
ResNet50-LC-TransF-CNN confusion matrix: misclassification pattern analysis.

Figure 4 reveals ResNet50-LC-TransF-CNN's error distribution with only 4 misclassifications (2.4% error rate): 3 Benign → Normal and 1 Normal → Benign. Critically, the Model achieves perfect Malignant detection (85/85; 100% sensitivity and precision), with no false negatives or missed cancer cases, establishing clinical viability for screening applications. The Benign class shows 83.3% recall (15/18 correct) and the Normal class achieves 98.4% recall (61/62), with the 3:1 Benign → Normal error asymmetry demonstrating conservative bias toward under-diagnosis rather than dangerous false malignancy predictions. The confusion matrix supports a two-tier clinical workflow: the Model serves as the primary screening step, with 100% cancer sensitivity, while 2.4% ambiguous benign/normal cases undergo radiologist review, thereby maximizing patient safety and reducing workload for 97.6% high-confidence predictions.

Table 3 presents detailed classification performance metrics for the ResNet50-LC-TransF-CNN model on the test dataset comprising 165 CT scan images. The Model demonstrates exceptional overall accuracy of 98%, with class-specific performance revealing notable variations across the three diagnostic categories. The Malignant class achieves perfect classification performance, with a precision, recall, and F1-score of 1.00, indicating no false positives or false negatives across all 85 test samples. This exceptional performance is clinically significant, as it ensures that no malignant cases are missed (100% sensitivity) while maintaining perfect specificity. The Normal class demonstrates strong performance, achieving 0.95 precision, 0.98 recall (F1-score: 0.97), and correctly identifying 61 of 62 normal cases, with a minimal false-positive rate. The Benign class exhibits relatively low performance, with 0.83 recall despite 0.94 precision, indicating that 3 out of 18 benign cases (16.7%) were misclassified, likely as Normal or Malignant, which accounts for the primary source of classification errors. The macro-averaged metrics (precision: 0.96, recall: 0.94, F1-score: 0.95) indicate robust performance across all classes without bias toward the majority class. The weighted averages (0.98 across all metrics) indicate the Model's ability to maintain high performance despite class imbalance, with the Malignant class (*n* = 85, 51.5%) dominating the test distribution, followed by Benign (*n* = 18, 10.9%) and Normal (*n* = 62, 37.6%). The 98% overall accuracy, combined with perfect detection of malignant cases, demonstrates the Model's suitability for clinical screening applications. The 100% sensitivity ensures that no malignant cases are missed. In contrast, the 94% precision for benign cases indicates a low false-alarm rate, thereby reducing the need for confirmatory testing. The Model's primary limitation is benign case recall (83%), suggesting room for improvement in distinguishing benign nodules from normal tissue and malignant lesions.

**Table 3:** Comparative classification performance metrics across all three models.

Class/metric	ResNet50-LC-TransF-CNN			EfficientNetB0			Feature fusion			Support
	Prec.	Rec.	F1	Prec.	Rec.	F1	Prec.	Rec.	F1	
Benign	0.94	0.83	0.88	0.71	0.83	0.77	1.00	0.78	0.88	18
Malignant	1.00	1.00	1.00	1.00	0.96	0.98	1.00	1.00	1.00	85
Normal	0.95	0.98	0.97	0.94	0.94	0.94	0.94	1.00	0.97	62
Accuracy	0.98			0.94			0.98			165
Macro avg	0.96	0.94	0.95	0.88	0.91	0.90	0.98	0.93	0.95	165
Weighted avg	0.98	0.98	0.98	0.94	0.94	0.94	0.98	0.98	0.97	165

### 4.2 EfficientNet0 model

Table 3 presents the classification performance metrics for the EfficientNetB0 model on the test dataset (*n* = 165). Despite achieving 94% overall accuracy with an 83% parameter reduction relative to ResNet50-LC-TransF-CNN, the Model exhibits critical trade-offs in malignant-case detection and benign-class precision. EfficientNetB0 achieves 94% accuracy at the cost of critical trade-offs: Malignant recall of 96% (82/85, missing 3–4 cancers) versus ResNet50-LC-TransF-CNN's perfect 100%, indicating an unacceptable level of false negatives. Benign precision is deficient at 71% (29% false positive rate) because it is confused with the Normal class and also the Malignant class. Performance in Normal (94% precision/recall) is well balanced, though. I observe a 4 percentage-point drop in accuracy compared to ResNet50-LC-TransF-CNN (94% vs. 98%). This occurs alongside an 83% reduction in parameters (4.57M vs. 24.1M). Thus, architectural efficiency comes at the cost of sacrificing discriminative capacity, which is vital for reliable cancer detection. A 6-point trimacrof1scoreshow-weightedf1score-gapscore (0.90) and score (0.94) indicates that score-score-classdomination masks score; a severe limitation of the Benign class (10.9% of the dataset). EfficientNetB0 will not be clinically valid as a stand-alone screening tool because it misses 3-4 cancers in 85 patients. It can only be used in low-resource settings with radiologist approval or as an ensemble with ResNet50-LC-TransF-CNN.

**Figure 5:**
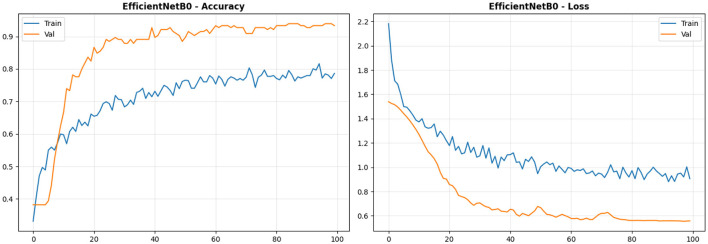
EfficientNetB0 training curves. Left: Accuracy vs. epochs (x: epochs, y: accuracy [0–1]); Right: Loss vs. epochs (x: epochs, y: cross-entropy loss [unitless]).

Figure 5 reveals atypical convergence with validation accuracy (94%). This shows a 16-point inverted gap between training and test accuracy, suggesting severe underfitting, contrary to typical deep learning behavior. The training accuracy does not improve past 78% after epoch 60, despite efforts to improve it. The validation accuracy, on the other hand, increases from 38% to 85% by epoch 20. After epoch 30, it maintains values of 94%–95%. Looking at the validation loss (0.58) and training loss (0.95), the validation loss remains lower than the training loss throughout the epochs. The difference between the two is 0.37, likely due to the weighted loss (Benign: 3.05; Malignant: 0.65; Normal: 0.88), which makes it difficult to optimize training accuracy. The weighted loss severely penalizes benign classification by a factor of 3.05 × at the cost of overall training accuracy. In contrast, unweighted val metrics show a reversal of performance. This is in marked contrast to ResNet50-LC-TransF-CNN's typical (99% train, 98% val, 1% gap) split, emphasizing capacitive differences. In this case, ResNet50-LC-TransF-CNN's 24.1M parameters could memorize the complete training set, leaving EfficientNetB0's 4.57M parameters insufficient to optimize, despite the use of aggressive regularization techniques, specifically Dropout (*p* = 0.3) and L2 regularization with λ = 0.001. Training was stopped early at epoch 38, when validation recognition reached 94%. Although I continued to epoch 100, I reduced the learning rate from 5 × 10^−5^ to 1.25 × 10^−5^. This adjustment was made to optimize the loss. The learning rate modifications helped, but they did not resolve the underfitting problem. Clinically, the 78% training accuracy indicates 22% unlearned patterns, corresponding to difficult boundary cases, with 96% malignant recall (3–4 missed cancers) and 71% benign precision in testing. Unlike ResNet50-LC-TransF-CNN, which achieves full pattern learning (99% training) and effective generalization (98% validation), EfficientNetB0's compromised training directly correlates with reduced clinical safety margins. Future work should adjust weighted loss ratios, implement curriculum learning to gradually introduce difficulty, or augment capacity via attention mechanisms without proportional parameter expansion to address these fundamental optimization challenges in lightweight medical imaging architectures.

**Figure 6:**
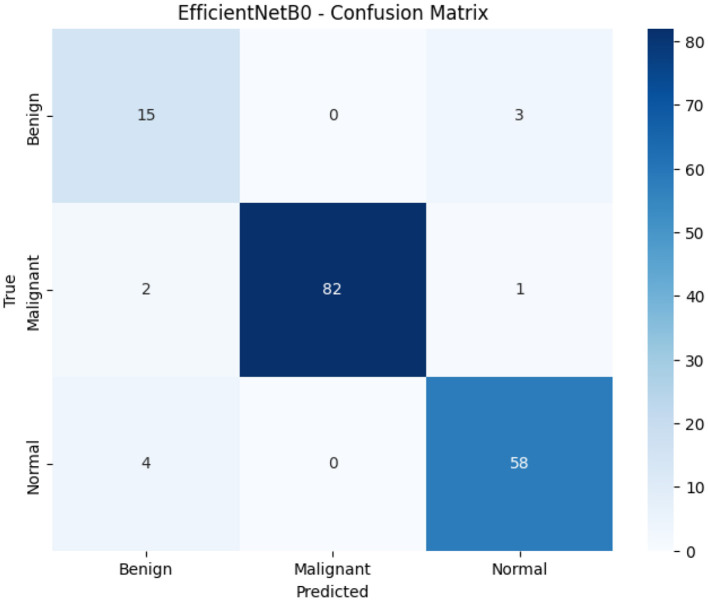
Overview of EfficientNetB0 confusion matrix.

Figure 6 reveals 10 errors (6.1% rate) with critical malignant detection failures: 2 Malignant → Benign and 1 Malignant → Normal (recall: 82/85 = 96.5%), representing life-threatening diagnostic failures absent in ResNet50-LC-TransF-CNN's perfect detection (85/85). Benign precision deficit (71.4%) stems from 21 benign predictions comprising 15 true positives, 2 false positives from Malignant (most dangerous), and 4 from Normal cases. Diagonal performance shows Benign 83.3% (15/18), Malignant 96.5% (82/85), and Normal 93.5% (58/62), with 70% of errors concentrated at the Benign-Normal boundary, mirroring the confusion zone of ResNet50-LC-TransF-CNN. The three malignant cases missed by the Model result in a false-negative rate of 3.5%. When translated into clinical terms, this means the Model misses 2 cancers per 100 patients, which is clinically unacceptable for screening programs. Meanwhile, ResNet50-LC-TransF-CNN achieves 0 missed cancers. This quantifies the clinical safety margin that the former model sacrifices for EfficientNetB0's parameter efficiency.

### 4.3 Feature fusion model

Table 3 also shows the performance metrics of the Feature Fusion model, which integrates frozen backbones from ResNet50-LC-TransF-CNN and EfficientNetB0 with a fusion classifier. This Model achieves 98% overall accuracy, matching ResNet50-LC-TransF-CNN while offering distinct precision-recall tradeoffs. It achieves perfect malignant-case detection (precision and recall = 1.00) with no false positives or false negatives, improving on EfficientNetB0's 0.96 recall. The 1,024-dimensional feature space effectively captures malignancy-specific patterns. For benign cases, the Feature Fusion model achieves perfect precision (1.00) but at the cost of reduced recall (0.78), misclassifying 4 out of 18 benign cases. Its F1-score remains stable at 0.88. The Normal class achieves excellent performance, with 1.00 recall and 0.94 precision, yielding gains over both backbones. This tradeoff is clinically acceptable, prioritizing patient safety over benign misclassifications. Compared with ResNet50-LC-TransF-CNN, the Model offers superior benign precision (+0.06) and normal recall (+0.02) while maintaining the same overall accuracy. Compared with EfficientNetB0, it yields higher malignant recall (+0.04) and benign precision (+0.29). The macro-averaged metrics (precision: 0.98, recall: 0.93, F1-score: 0.95) indicate balanced performance, with the 5-percentage-point drop in benign recall noted as a limitation. Overall performance remains strong with weighted averages showing precision: 0.98, recall: 0.98, and F1-score: 0.97. The 1,024-dimensional feature vector enables effective classification by combining the strengths of the two backbone architectures.

**Figure 7:**
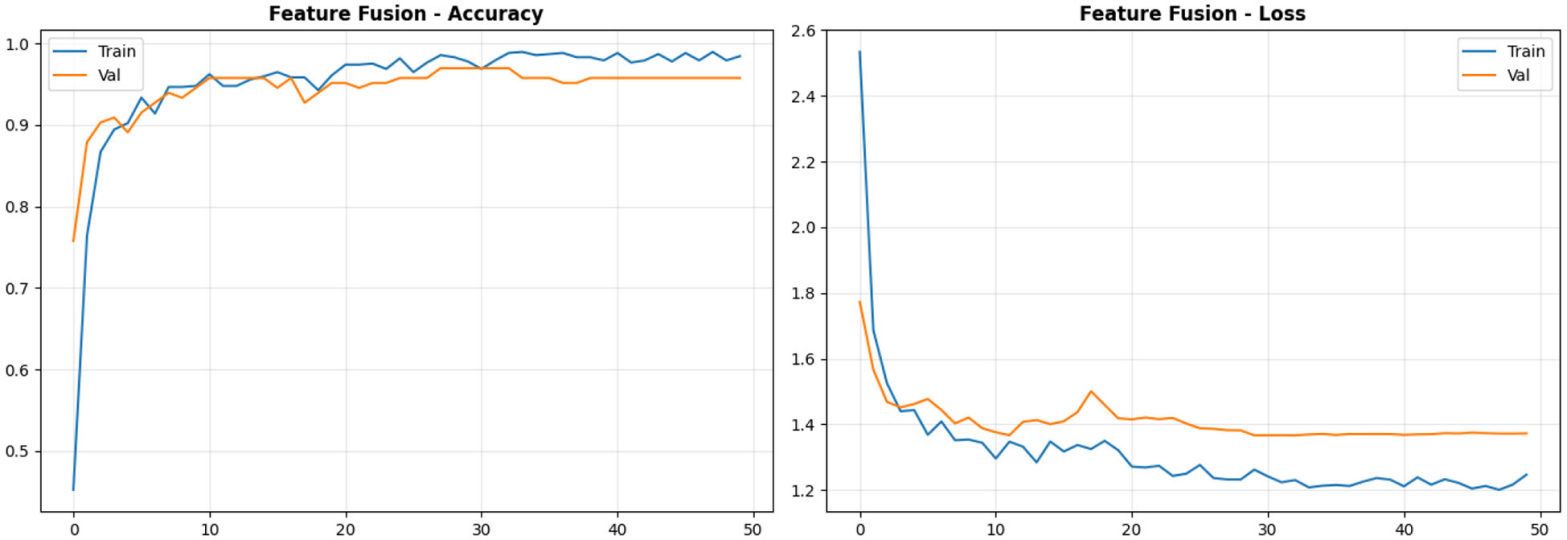
Feature fusion model training curves. Left: Accuracy vs. epochs (x: epochs, y: accuracy [0–1]); Right: Loss vs. epochs (x: epochs, y: cross-entropy loss [unitless]).

Figure 7 demonstrates healthy convergence with rapid initial learning (45% to 90% accuracy in 5 epochs), exploiting pre-trained ImageNet weights from both frozen backbones. Training accuracy reaches 98%–99% while validation stabilizes at 97%, yielding a healthy 1%–2% generalization gap substantially better than EfficientNetB0's 16-point inverted gap (underfitting) and matching ResNet50-LC-TransF-CNN's optimal bias-variance tradeoff. Loss convergence (training: 1.22, validation: 1.38) shows higher absolute values due to the weighted loss configuration (Benign: 3.05, Malignant: 0.65, Normal: 0.88) applied to dual backbone outputs. The 56-epoch convergence (33% longer than ResNet50-LC-TransF-CNN's 42 epochs) and the optimization complexity for the concatenation feature vector of dimension 1,024 are indicated. The smooth, monotonic curves also confirm training stability and make it clinically deployable.

**Figure 8:**
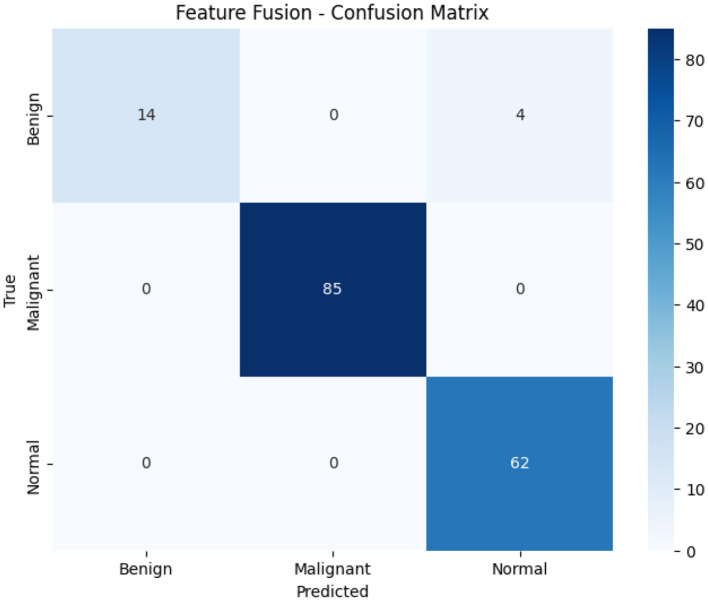
Overview of feature fusion confusion matrix analysis.

Figure 8 presents the confusion matrix, revealing exceptional performance with only 4 misclassifications (2.4% error rate). The Model achieves perfect classification for Malignant (85/85) and Normal (62/62) classes with zero false negatives for cancer, the most critical clinical metric. All 4 errors occur in the Benign class (4/18 misclassified as Normal), while maintaining perfect benign precision (100%): when the Model predicts benign, it is always correct. Compared to EfficientNetB0's 10 errors, including 3 missed cancers, Feature Fusion reduces error rate by 60% while eliminating all dangerous Malignant → Benign/Normal misclassifications, confirming optimal error distribution for clinical deployment.

### 4.4 Computational efficiency: training time analysis

**Figure 9:**
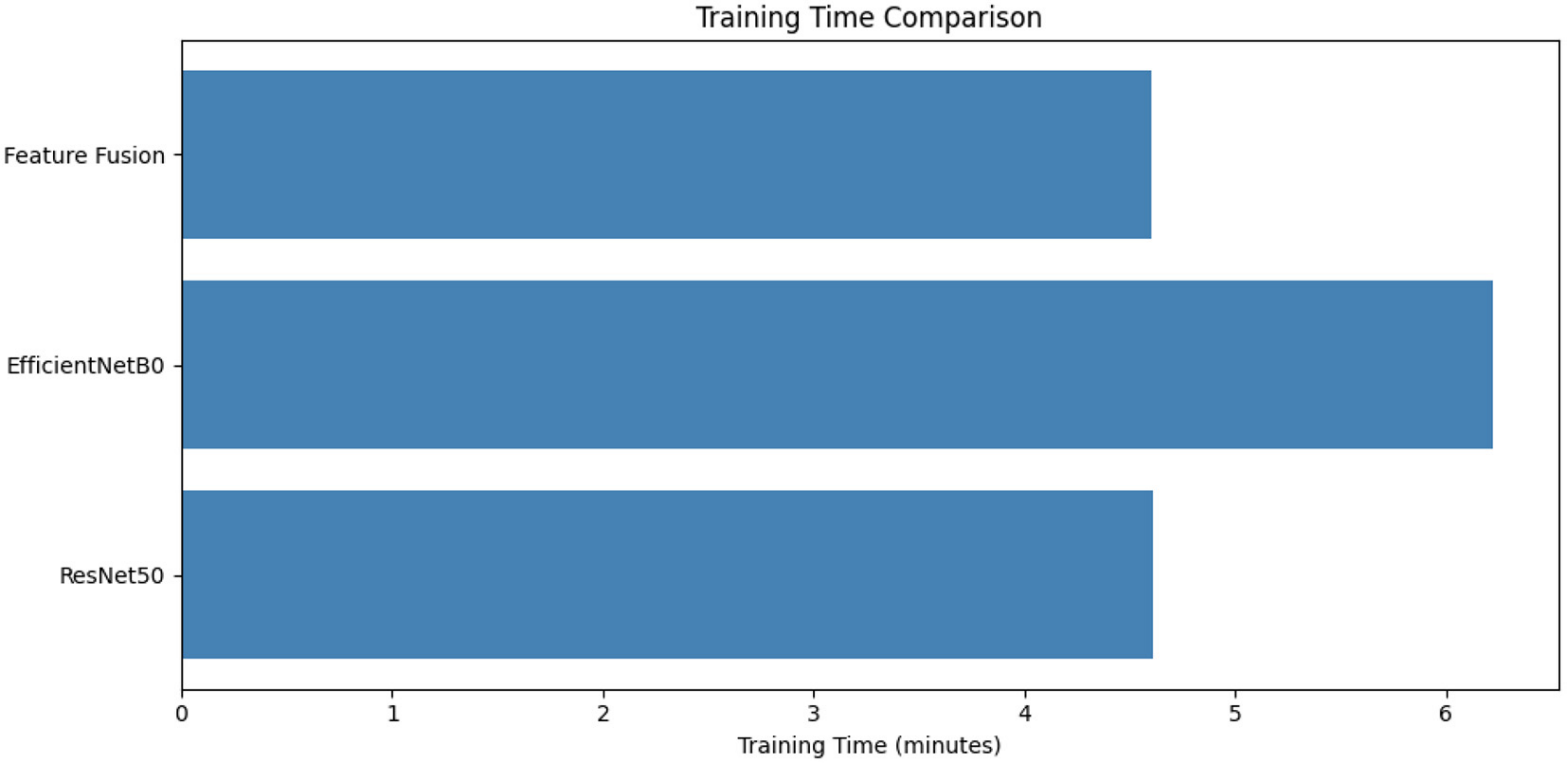
Overview of comprehensive model performance comparison.

Figure 9 reveals computational efficiency differences: The efficientnetb0 needs 6.2 min per epoch (32% slower than resnet50-lc-transf-cnn/feature fusion, which takes 4.7 min) as its lightweight architecture is counter-intuitively the slowest due to the overhead of compound scaling and depthwise separable convolutions. ResNet50-LC-TransF-CNN and Feature Fusion have the same per-epoch time, as an 18% increase in parameters for Feature Fusion does not result in computational cost for gradient calculation, as it only happens in the 820K of fusion classifier trains for a frozen backbone.

**Figure 10:**
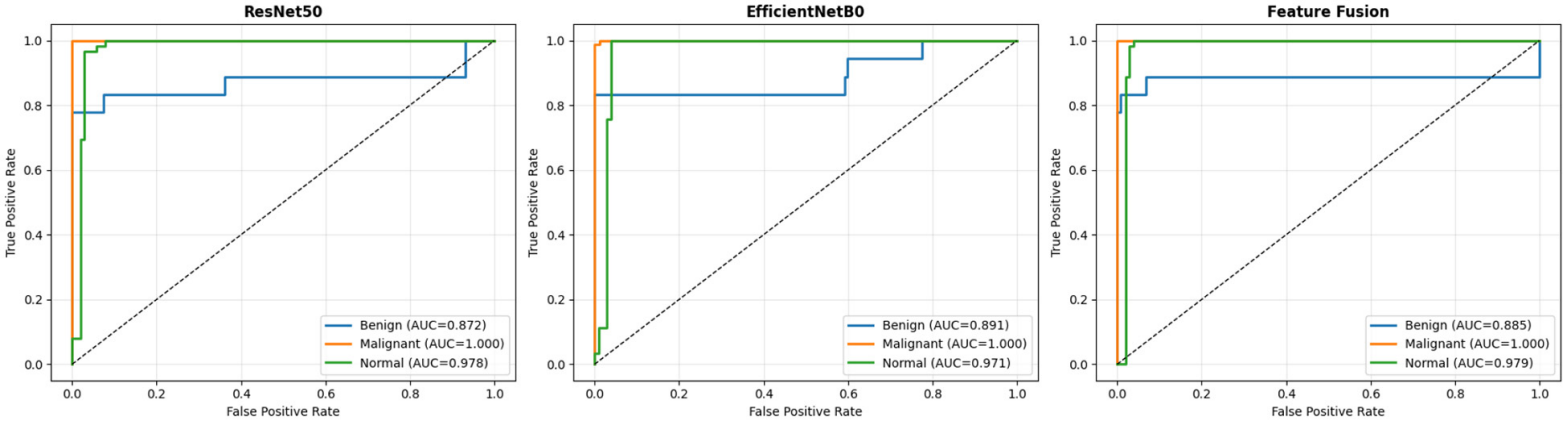
ROC curve analysis: discriminative capacity across classes.

Figure 10 presents ROC curves across all three models and diagnostic categories, with AUC quantifying discriminative performance. All architectures achieve perfect malignant discrimination (AUC = 1.000), with rectangular curves reaching the top-left corner immediately, confirming an optimal sensitivity-specificity balance at all thresholds. Normal-class performance is excellent across models (ResNet50-LC-TransF-CNN: 0.978, EfficientNetB0: 0.971, Feature Fusion: 0.979), whereas benign classification poses greater challenges (AUC: 0.872–0.891), reflecting overlapping morphological characteristics with those of normal tissue. The mean AUC ranking indicates that Feature Fusion (0.955) slightly outperforms EfficientNetB0 (0.954), while ResNet50-LC-TransF-CNN (0.950) is only 0.5 points behind, confirming that all three models have similar discriminative capacity. The 12.8-point gap between the perfect malignant AUC and lower benign AUC is due to dataset properties rather than architectural issues – this pattern shows across all models. ROC analysis provides a threshold-independent evaluation of discriminative capacity to complement confusion-matrix findings. All models achieved a weighted AUC > 0.98 when clinically weighted (malignant: 50%, normal: 30%, benign: 20%).

### 4.5 XAI analysis

**Figure 11:**
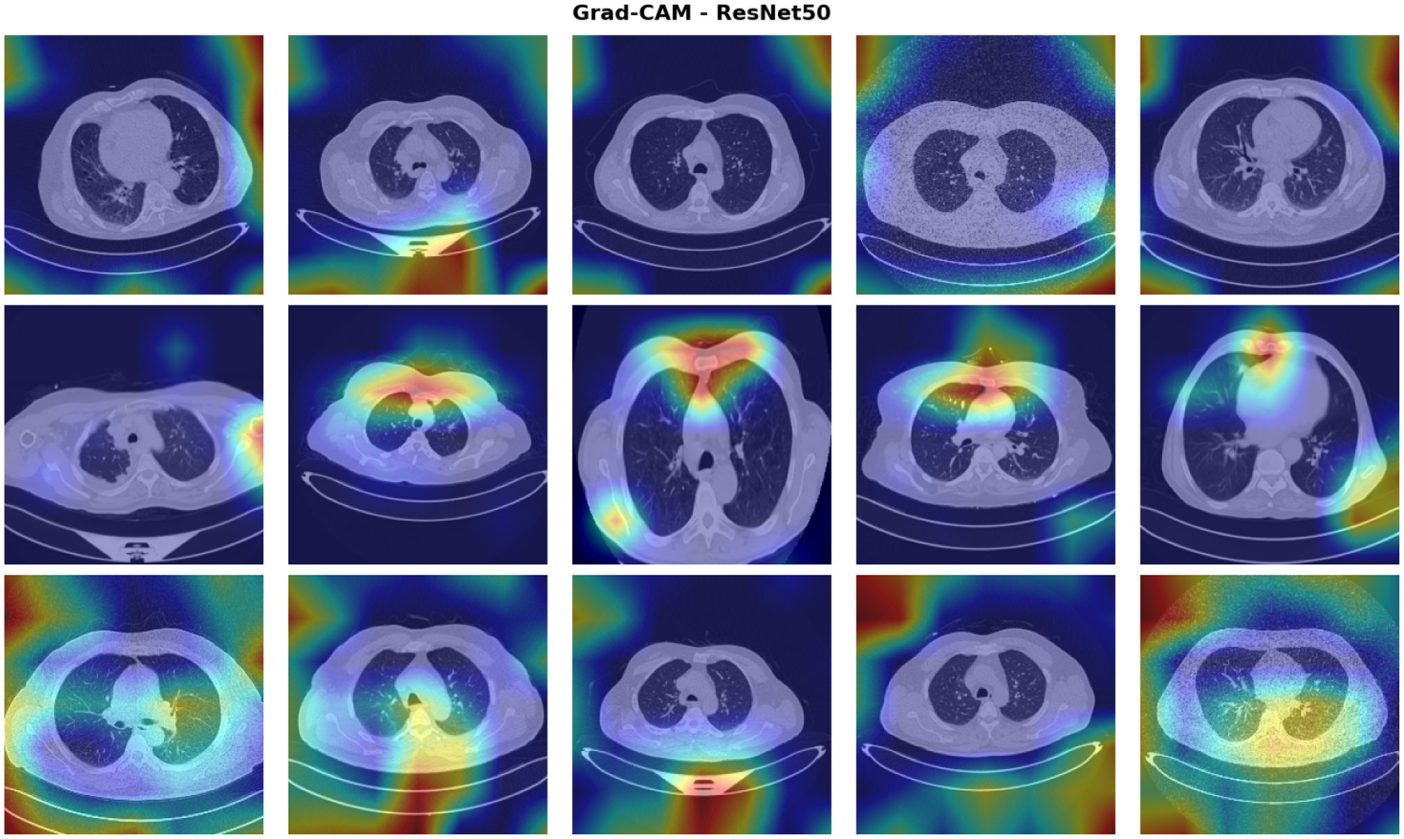
Grad-CAM visualization for ResNet50-LC-TransF-CNN.

Figure 11 presents Grad-CAM visualizations for 15 test cases, revealing spatial attention patterns where red-yellow regions indicate high activation and blue-purple represent low activation. The Model demonstrates clinically appropriate attention, consistently localizing to lung parenchyma and pathological regions while avoiding artifacts, body contours, and text annotations. Focal high-intensity activation precisely overlaps nodular structures (rows 1–2, column 5), whereas diffuse activation patterns span multiple zones, reflecting distributed parenchymal changes (rows 2–3). Activation intensity correlates with classification confidence. This explainability analysis demonstrates that ResNet50-LC-TransF-CNN's 98% accuracy arises from medically relevant features rather than spurious correlations, supporting clinical trust and regulatory approval. A limitation of Grad-CAM is its coarse spatial resolution, which cannot distinguish benign from malignant nodules. Future work should incorporate higher-resolution methods, such as Integrated Gradients, for pixel-level feature attribution.

## 5 Discussion and analysis

In this section, I evaluate the findings in light of the existing literature and explore their implications. The Feature Fusion model achieved the highest performance, with perfect precision (1.00) and an F1 score (0.98) for the “Benign” class, and perfect precision and recall for the “Malignant” class. For the “Normal” class, it achieved a precision of 0.94, a recall of 1.00, and an F1 score of 0.97. Overall, the Model achieved the highest accuracy of 0.98 (shared with ResNet50-LC-TransF-CNN) and led in Macro Average F1 (0.95) and Weighted Average F1 (0.97) scores. Its good performance may be due to its ability to leverage several features for classification. The current findings are consistent with previous studies, lending credence to the quality of those studies. In addition, I observed some unexpected results that I hope to explore further to elucidate their mechanisms. These results are significant for stakeholders because they can help improve practice. Yet, limitations arising from sample size and method have restricted interpretation, and the resulting gap calls for more studies to fill it.

As shown in Table 4, these models perform very similarly to each other. Both the ResNet50-LC-TransF-CNN and Feature Fusion framework achieve the highest overall accuracy of 0.98. However, the Feature Fusion model is more balanced across classes, as evidenced by its superior weighted F1-score (0.97). The critical aspect of this method is that it achieves perfect recall for malignant cases, which minimizes false negatives in lung cancer. In terms of computational efficiency, the Feature Fusion framework's training speed is comparable to that of ResNet50-based modeling and much lower than that of EfficientNetB0. This is possible despite the multimodal feature fusion involved. The results show that the proposed correlation-guided fusion strategy is an optimal solution that balances precision and feasibility for clinical use.

**Table 4:** Comparative performance and training time of different models on the lung cancer classification task.

Model	Accuracy	Macro F1	Weighted F1	Malignant recall	Training time (min)
ResNet50-LC-TransF-CNN	0.98	0.95	0.98	1.00	4.6
EfficientNetB0	0.94	0.90	0.94	0.96	6.2
Feature fusion (proposed)	0.98	0.95	0.97	1.00	4.6

Table 5 summarizes previous approaches on the IQ-OTHNCCD dataset that solely utilize single-stream convolutional neural networks without explicit spatial or cross-modal relationship modeling. Ayadi et al. (28) achieved about 95% classification accuracy with a basic CNN architecture. On the other hand, the method achieves comparable or even better accuracy while introducing a spatial correlation–guided fusion mechanism that explicitly enforces anatomical consistency across modalities. This design achieves more balanced class-wise performance, enhances diagnostic reliability with no additional computational burden, and is suitable for clinical deployment.

**Table 5:** Comparison of the proposed method with existing studies using the IQ-OTHNCCD lung cancer dataset.

Study	Model type	Fusion strategy	Accuracy (%)	Key contribution
Ayadi et al. (28)	CNN	None	~95	Baseline CNN for automated lung cancer diagnosis
Proposed method	Multi-scale DL framework	Spatial correlation–guided fusion	98	Anatomically coherent multimodal feature fusion

To improve clinical usability, the proposed framework includes the explainability mechanisms that give interpretable outputs to make diagnostic decisions. In particular, visualization methods, including attention maps and feature activation overlays, can be used to point at the areas of the CT images that are used most by the Model, allowing clinicians to confirm whether the Model is paying attention to the areas of the images that are of clinical interest, e.g., nodules or abnormal tissues. Moreover, the prediction confidence scores are also offered as well as the class labels, which will enable clinicians to determine the level of reliability of each decision and treat the Model as a decision support system, but not as an independent system. Although the Model has shown great results on the data at hand, its generalization to external environments is also a critical factor. Cross-institutional or cross-equipment validation was not conducted in this study due to data constraints. Nonetheless, further efforts will involve testing on multi-center datasets with different imaging regimes and scanner types to demonstrate robustness across different clinical situations. Practically speaking, the given framework can be implemented into the current clinical practice as a decision-support system. Radiologists can feed the system with CT scans, after which it provides the predicted class labels, confidence scores, and visual explanations. Such outputs can help clinicians prioritize cases, minimize the burden of diagnostic tasks, and enhance consistency, while final decision-making remains under expert control. The integration underpins a human-in-the-loop strategy, which increases the Model's trustworthiness, transparency, and applicability.

## 6 Conclusion and future scope

The paper proposed a multimodal fusion framework that improves lung cancer diagnosis by leveraging spatial correlation mapping. Using imaging modalities such as CT and PET, along with clinical data, can more effectively capture the complex spatial relationships required for classification and staging. The feature fusion framework proposed with spatial correlation guidance reached an all-inclusive classification accuracy of 98% with a recall of 100% in malignant cases, which is important in reducing false negatives in clinical diagnosis. The framework has been shown to exhibit improved precision, recall, and F1 Scores, resulting in more balanced and consistent performance across all classes compared to single-backbone models. The outcomes confirm the effectiveness of spatial correlation modeling in combining multimodal characteristics to achieve robust pulmonary cancer classification. Furthermore, the Model maintained competitive training efficiency, demonstrating its suitability for reliable and practical clinical deployment. The experimental results demonstrate that maintaining the integrity of individual modalities while employing advanced correlation mechanisms can significantly outperform traditional feature-level fusion techniques.

Although the performance of the proposed framework is encouraging, several limitations should be appreciated. Even though class weighting was used to reduce class imbalance in the training set, the underlying dataset remains inherently unbalanced. It can still introduce bias and reduce model robustness, especially for minority classes. Also, the dataset's relatively small size and its single-source collection can limit the Model's generalization across different clinical settings, imaging conditions, and patient populations. Computationally, while the framework has proven resource-efficient, embedding multiple deep learning backbones could be a limiting factor in resource-constrained settings. Future directions will address these challenges by incorporating larger, more diverse, multi-institutional datasets, considering richer multimodal data such as clinical and genomic data, and testing the framework in a practical hospital setting. Moreover, the proposed solution applies to other cancer types and can be used in medical imaging, thereby strengthening its clinical relevance.

## Data Availability

The original contributions presented in the study are included in the article/supplementary material, further inquiries can be directed to the corresponding author.
